# Molecular foundations of collagen triple helical assembly: the central role of prolyl-4-hydroxylation

**DOI:** 10.1042/BCJ20253467

**Published:** 2026-01-08

**Authors:** Ashutosh Joshi, Bhaskar Mondal, Trayambak Basak

**Affiliations:** 1 School of Biosciences and Bioengineering, Indian Institute of Technology Mandi, Mandi, Himachal Pradesh, 175075, India; 2 School of Chemical Sciences, Indian Institute of Technology Mandi, Mandi, Himachal Pradesh, 175075, India

**Keywords:** collagen, thermal stability, 4-hydroxyproline, pyrrolidine ring pucker, peptide bond isomerization, charge–transfer interactions

## Abstract

Collagen, the principal structural protein of the extracellular matrix, has been pivotal in the evolution of multicellular life. Among its posttranslational modifications (PTMs), prolyl-4-hydroxylation stands out as the most conserved and functionally critical. This hydroxylation not only facilitates polyproline II-like helicity in single collagen strands but also underpins the stability and integrity of the triple helix. In this review, we summarize the molecular foundations of collagen assembly, emphasizing both thermodynamic and stereo-electronic contributions of 4-hydroxyproline (4-Hyp). Insights from collagen-mimetic peptides are highlighted to elucidate main-chain torsional preferences, the positional dependence of proline ring puckering, and the correlation between 4-Hyp and enhanced thermal stability. Parallel discussions explore stereo-electronic effects revealed through quantum chemical studies, particularly charge–transfer interactions that modulate pyrrolidine ring conformation and peptide bond geometry. Finally, we summarize advances from periodic quantum mechanical calculations that quantify interstrand binding energies across different tripeptide motifs. Together, these findings provide a molecular-level perspective on how prolyl-4-hydroxylation has shaped the unique stability and evolution of collagenous helicity.

## Introduction

Collagen, an abundant extracellular matrix (ECM) protein, is *a priori* for the evolution of life from the unicellular to multicellular organisms [1]. Previous studies have suggested that the genes encoding for the monomeric unit of collagen, the –Xaa–Yaa–Gly– (XYG) triplet, originated early in the evolutionary tree, with evidence of their presence in protists. However, type I collagen, which is predominant in human bone or skin, is believed to appear at a later stage in the course of evolution, specifically within the kingdom Animalia [1]. To date, 28 types of collagen have been identified and classified into 7 categories in humans based on their structural and functional characteristics. All of them comprise a typical triple helical domain [2]. Previous studies observed distinct diffraction patterns from collagen samples than typical α-helices, deciphering the coiled-coil structure of a triple helical domain of a collagen molecule [3–9]. Studies have demonstrated that the three left-handed polyproline type II (PPII)-like single strands adopt the right-handed helical conformation around a central axis and assemble into a trimeric protein, i.e. collagen triple helix [5,8]. Additionally, the structural investigations revealed staggering of individual strands by one amino acid and suggested the regularity of Gly being every third residue in the repetitive XYG triplet motif of a strand [5,9–12]. These two attributes play significant roles in the assembly of the collagen triple helix. Staggering of strands is crucial to form lateral hydrogen bonds (perpendicular to the central axis) as it aligns Gly residues of one strand laterally to amino acid residues at the Xaa (X) of the adjacent strand. Consequently, the amide moiety (–(CO)NH) associated to the peptide bond of Gly residues acts as a donor and the carbonyl (–CO) associated to the peptide bond of amino acid residues at X as a recipient of hydrogen bonds. These interstrand hydrogen bonds hold the triple helix and confer stability [5,7–9]. Moreover, Gly accommodates favorably in the vicinity of the central axis owing to its short side chain (–H). Consequently, individual strands observe less steric hindrance while wrapping around the central axis to assemble into a triple helix [5,8].

Along with the structural investigations, the analysis of amino acid sequences substantiated the prevalence of tripeptide motif in the form of XYG and demonstrated the frequency of amino acid at the X and Yaa (Y) positions. One of the earlier studies suggested that one-third of the amino acids in collagen from a bovine tissue sample was hydroxyproline [13]. Later, it was proposed that X and Y positions predominantly accommodate proline (Pro) and 4-hydroxyproline (4-Hyp), respectively [10,11]. From amino acid analyses of vertebrates’ and invertebrates’ collagens, it was observed that ~33%, ~10–12%, and ~6–10% of the sequence accounted for Gly (G), Pro (P), and 4-Hyp (O^4^) residues, respectively [14]. Moreover, in the human fibrillar and non-fibrillar collagens, –Pro–4-Hyp–Gly– makes the most abundant tripeptide motif [15]. The larger content of Pro and 4-Hyp due to the highest frequency tripeptide motif –Pro–4-Hyp–Gly– demonstrates a positive correlation with the thermal stability of the collagen triple helix [12,16]. More insights on such a sequence–structure relationship would have been gained with a full-length collagen crystal structure. To date, this has remained unachieved due to the complexity, large size, and insoluble nature of the collagenous triple-helical molecules. However, the challenge was addressed with the synthesis of collagen model peptides (CMPs) through the host–guest peptide strategy [17–19]. CMPs with repeats of a pure tripeptide motif, such as (PPG)_10_ and (PO^4^G)_10_, forming triple helices, were crystallized and investigated for establishing the correlation between 4-Hyp and thermal stability [18,20–25]. Earlier studies investigating the crystallized PO^4^G-comprising CMP suggested that 4-Hyp causes the elevation in the melting temperature of collagen due to the increased number of hydrogen bonds formed between the hydroxyl (–OH) group of 4-Hyp and the carbonyl (–CO) group in the peptide backbone [18,26]. However, this notion was overruled by Raines and co-workers after analyzing the melting temperature of a CMP comprising –Pro–4-Flp–Gly– (PFlG) with establishing the fact that the electron-withdrawing group (EWG) such as –OH strictly prefers a specific pyrrolidine ring and peptide bond conformations due to the inductive effect and thereby pre-organizes the single strands to assemble into a stable triple helix [27]. The preferences of such conformations required for stable CMPs using host–guest peptides were used to establish nature’s selection of prolyl-4-hydroxylation as a typical abundant collagen posttranslational modification (PTM). Studies highlighting the preferences of the pyrrolidine ring and peptide bond with several substituents at 4-C of Pro are discussed below (*vide infra*).

**Figure 1:**
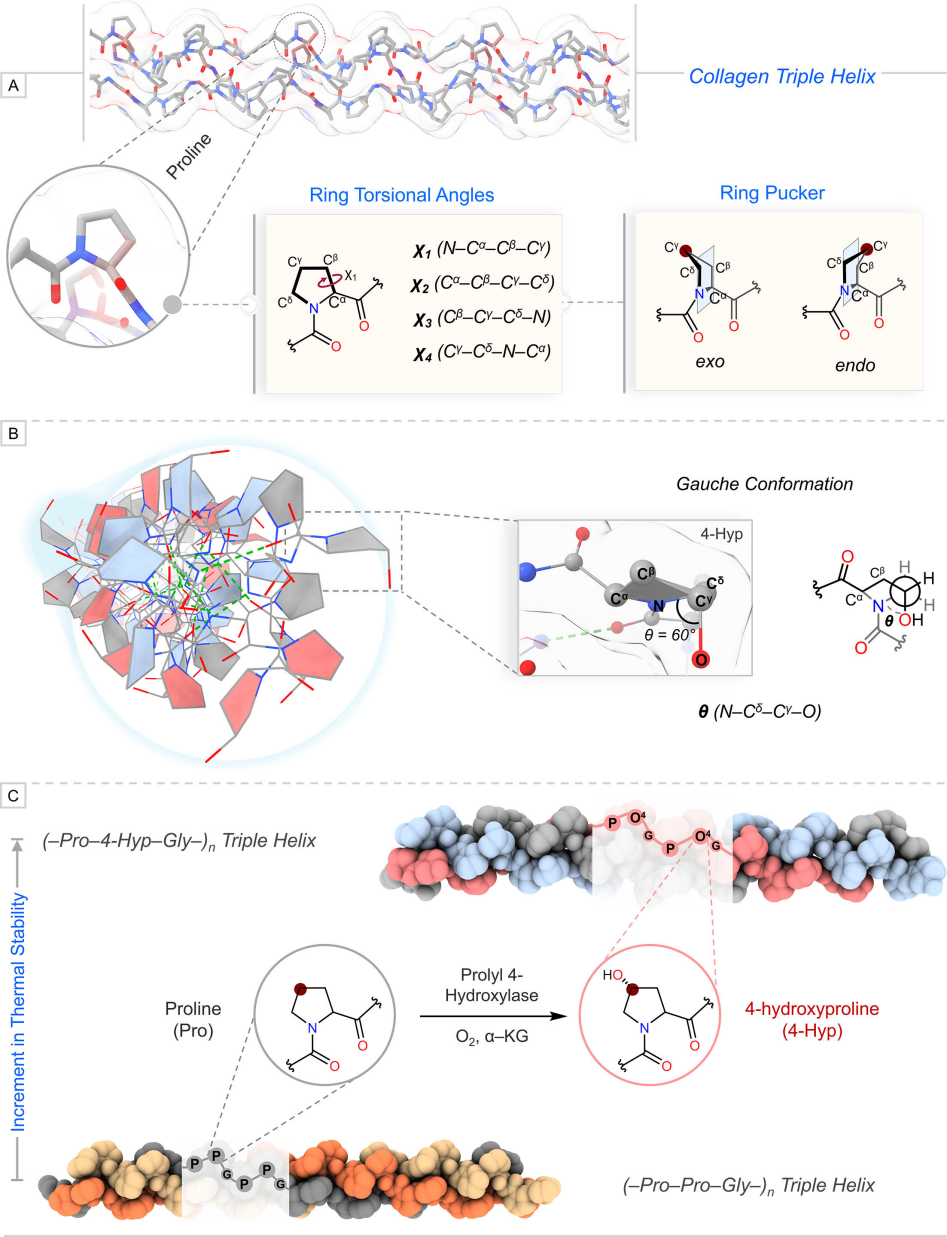
(A) Definition of ring torsional angles and two ring puckers that can be adopted by the pyrrolidine ring. (B) The left side represents the collagen triple helix, having 4-hydroxyproline (4-Hyp) at Y, pointing out toward the viewer, which has three strands in which pyrrolidine rings are colored with gray, blue, and red. The green dashed line represents the interstrand hydrogen bonding. The right side demonstrates the molecular model and chemical drawing for the gauche conformation exhibited in 4-Hyp. (C) The posttranslational 4-hydroxylation on proline at Y elevates the thermal stability of collagen.

The structural investigation of a CMP comprising 4-Hyp at Y in the tripeptide motif suggested the crucial role of geometrical parameters and stereo-electronic effects in preferring specific conformations by the pyrrolidine ring and peptide bond to stabilize collagen [22,27–31]. Studies on the influence of substituents on ring pucker and peptide bond conformations using crystallized structure of Pro and its derivatives advanced the understanding of their synchronized role in collagen stability [30,32]. Ring torsional angles, specifically χ_1_, govern the conformational preference of the pyrrolidine ring pucker. The higher negative value of χ_1_ enforces the pyrrolidine ring to conform into the *exo* ring pucker, whereas being positive favors the *endo* pucker (Figure 1A) [32,33]. Generally, the intrinsic conformational preference of Pro is *endo*; however, it favors *exo* at the Y position in collagen [22]. The favorability of *exo* ring pucker for 4-Hyp at Y is further enhanced by the gauche effect due to –OH (Figure 1B). Consequently, a CMP having prolyl-4-hydroxylation at Y demonstrates a higher thermal stability than the CMP that has an unmodified Pro (Figure 1C) [27]. Collectively, the prolyl-4-hydroxylation modification in collagen, catalyzed by the enzyme prolyl-4-hydroxylase inside a cell, is essential to maintain its structure and stability.

The analysis of the occurrence of XPG motifs in human COL1A1 revealed that PPG has the highest frequency of occurrence, which is 36.2% [34]. However, COL1A1 also comprises other XPG motifs in which the X position can be occupied by amino acids other than Pro. The influence of the amino acid at the X position on prolyl-4-hydroxylation at Y indicates that positively charged or polar uncharged residues at X preferentially support P4HA1 activity, whereas negatively charged residues at X favor P4HA2 specificity [34]. Studies analyzing the melting temperature of host–guest peptides containing XO^4^G motif demonstrated that replacing Pro at the X position reduces the thermal stability of collagen, with aromatic amino acid residues and glycine showing largest destabilization [35,36]. Although the influence of amino acids at the X position on the thermal stability of collagen has been explored, the alteration caused by replacing Pro at X on the stereo-electronic effects of 4-Hyp stabilizing collagen has remained elusive. In collagen, there are XYG motifs in which either X/Y or both are occupied by amino acids other than Pro or 4-Hyp. Several studies have been conducted to understand the contribution of such motifs to collagen structure and stability. Initially, Brodsky and co-workers demonstrated that the triple helix containing arginine (R) at Y in the PYG motif is less stable only by ~0.1°C than that composed of PO^4^G [35]. Such stabilization probably occurs due to the side chain of arginine that could interact with the –CO of the peptide backbone [35]. Additionally, Hartgerink and co-workers and others have illustrated that the presence of charged amino acids plays a role in the interstrand salt–bridge interactions that dictate the favorability of heterotrimeric assembly of collagen [37–41]. However, whether these interactions influence stability through 4-Hyp or contribute to processes beyond collagen assembly remains to be determined.

**Figure 2:**
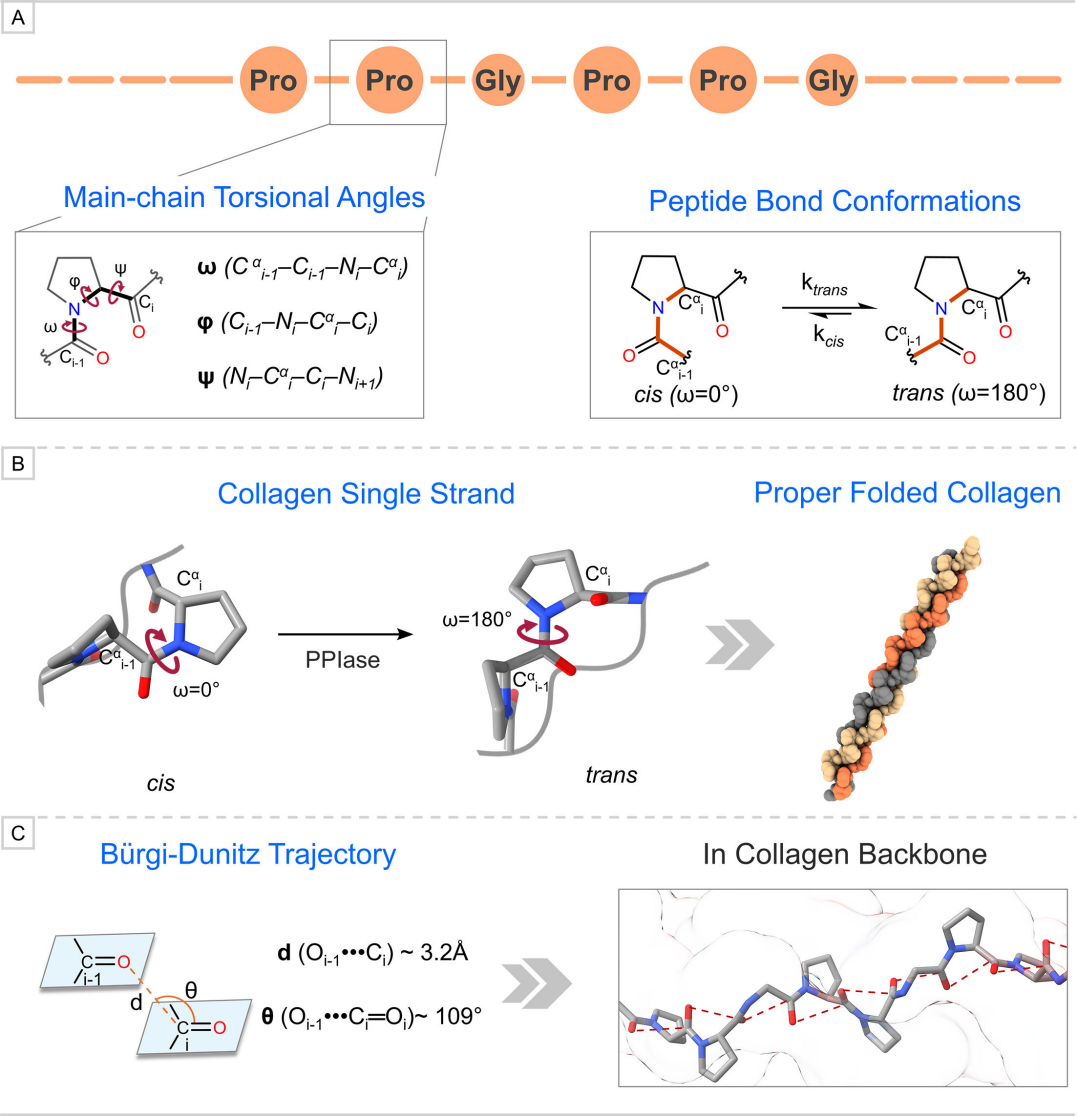
(A) Definition of main-chain torsional angles and isomerization of peptide bonds to obtain *cis* and *trans* conformations. (B) The isomerization of peptide bonds from *cis* to *trans* conformation was catalyzed by PPIase enzyme, which is essential for single strands to be folded into a triple helical structure. (C) Definition of the ideal values of Bürgi–Dunitz trajectory and its presence in the peptide backbone of collagen.

Another key characteristic of collagens established via exploring the CMP was the attainment of *trans* conformation by peptide bonds, which supports the earlier X-ray diffraction studies [4]. Due to the high content of Pro and 4-Hyp residues, main-chain torsional angles, φ and ψ, observe conformational restrictions in a single strand to attain PPII-like helicity (Figure 2A) [5,6,8,9,20,21]. The investigation of CMPs comprising 4-Hyp at Yaa revealed that the main-chain torsional angles φ and ψ attain values approximately −60 ± 7° and 150 ± 9°, respectively [42]. The conformational predominance of *trans* over *cis* by peptide bonds in individual strands is influenced by another main-chain torsional angle, i.e. ω (Figure 2A). An enzyme called prolyl-peptide *cis–trans* isomerase (PPIase) ensures that peptide bonds attain *trans* (ω≈ 180°) conformation by isomerizing *cis* (ω≈ 0°) peptide bond inside a cell (Figure 2B) [43,44]. The isomerization of peptide bonds is the rate-limiting step in collagen biosynthesis as *trans* conformation is essential for single strands to properly fold into a triple helix and its transportation to ECM (Figure 2B) [43,44]. Raines and co-workers discovered that the *trans* conformation of peptide bonds correlates with the optimal Bürgi–Dunitz trajectory for a non-covalent interaction (i.e. the n→π* charge–transfer interaction) to happen using crystallized structure of 4-Flp residue [30]. For this interaction to occur, the adjacent carbonyl groups must approach the ideal Bürgi–Dunitz trajectory, i.e. d (O_i−1_•••C_i_) approximately 3.2 Å and θ (∠O_i−1_•••C_i_═O_i_) near 109° (Figure 2D) [45]. In a polypeptide structure such as a single strand of collagen, this carbonyl–carbonyl interaction can have an additional structural effect on the structure (Figure 2D). Such advancement in the theoretical computational chemical field has enabled these discoveries to reveal the detailed stereo-electronic effects stabilizing the collagenous helicity.

Computational studies have significantly explored the underlying mechanism of stability provided by 4-Hyp using quantum chemical methods such as density functional theory (DFT). It has been computationally demonstrated that the –OH group at (4*R*)-position enforces the pyrrolidine ring to adopt the *exo* pucker as a result of the gauche effect, which is stabilized by a non-covalent interaction, i.e. the σ→σ* charge–transfer interaction [29,46,47]. Moreover, the attainment of *trans* conformation of peptide bonds in a single strand of collagen is a consequence of another non-covalent interaction, i.e. the n→π* charge–transfer interaction [29,30,46,48]. Additionally, our group has recently established that the elegant interplay between the switching of pyrrolidine ring pucker and peptide bond conformations is crucial for overall puckering and helical structural attainment [46]. Another quantum chemical method known as periodic DFT has also been utilized to investigate the stability of collagen provided by 4-Hyp at the full protein structure level, i.e. the triple helical structure [49].

In this review, we have summarized the current knowledge regarding the crucial role of the abundant PTM, i.e. 4-Hyp plays in the stability of collagen. We have touched upon both the experimental and computational investigations to provide detailed insights. We have outlined the utilization of CMPs to understand the role of 4-Hyp in maintaining the PPII helicity of single strands and their folding into a thermally stable collagen triple helix. In addition to this, we have also encapsulated experimental approaches to evaluate the stereo-electronic effects in collagen that are responsible for its stability. Further, we have extensively covered computational studies that have investigated the energetics of conformations of the pyrrolidine ring and the peptide bond, the electronic structure in the charge–transfer interaction, and the interstrand binding energy of collagen.

## Correlating melting temperature of collagen with conformational preference of pyrrolidine ring and peptide bond

As discussed above, CMPs have played a crucial role in elucidating the influence of the positional preference of 4-Hyp, including its specific pyrrolidine ring puckering and peptide bond isomerization, on the thermal stability. In this section, we summarize how CMPs have been employed to investigate the role of 4-Hyp and demonstrate that variations such as stereochemistry at 4-C of Pro, pyrrolidine ring puckering, and peptide bond isomerization contribute to the stabilization of triple helices. These stabilized triple helices, in turn, have the potential to be utilized in the development of specific scaffolds in biomedical research.

### Preferential attainment of pyrrolidine ring at Yaa

**Figure 3:**
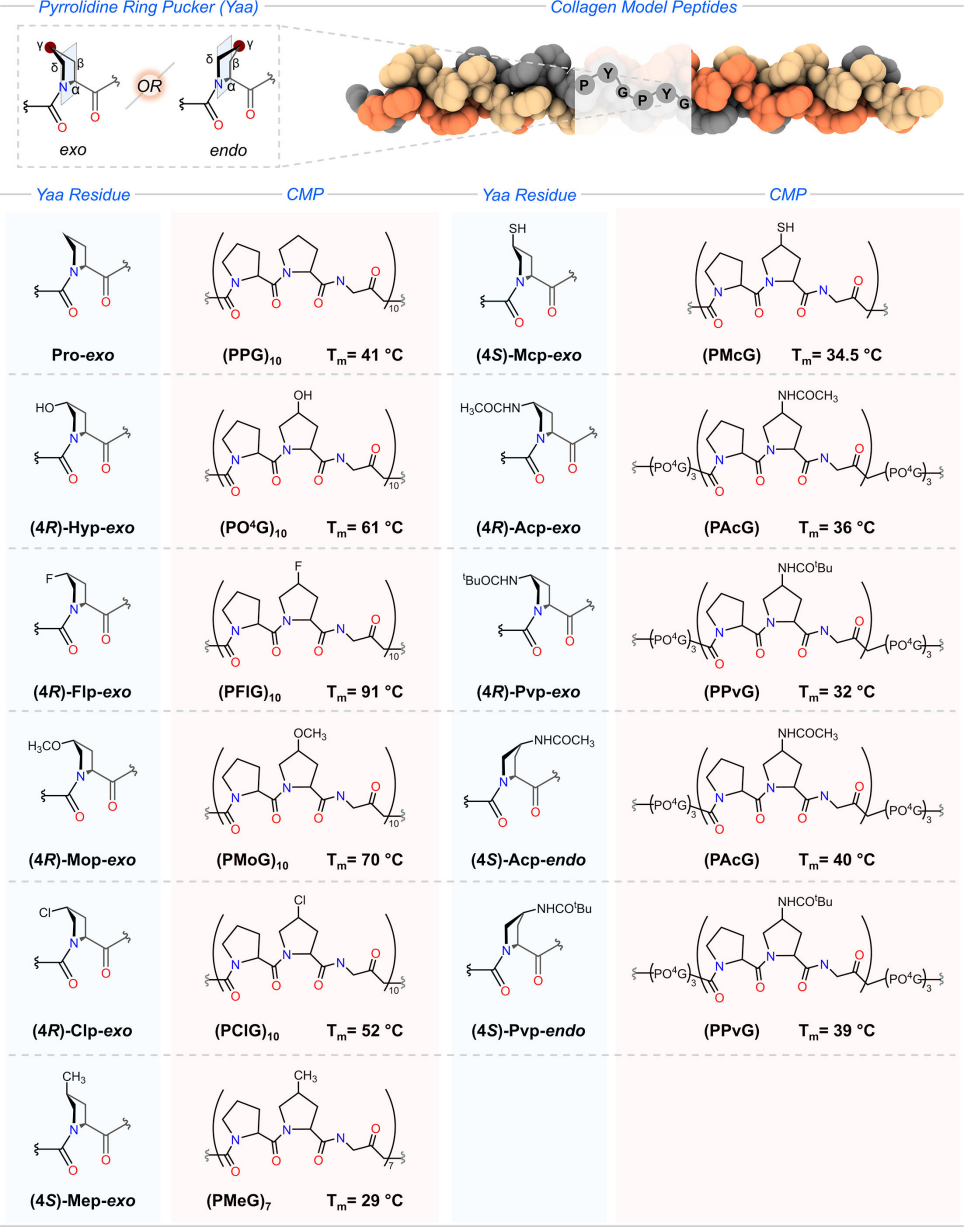
Top panel: The proline derivative can adopt any ring pucker while occupying Yaa position in collagen triple helix. Bottom panel: Proline derivatives at Yaa and corresponding collagen model peptides (CMPs) with melting temperature (T_m_).

In the repeating tripeptide motif of collagen, Pro or its derivatives occupying the Y position can adopt either *endo* or *exo* ring pucker, a conformation that is significantly influenced by substitutions at the 4-C position [18,20–22,25,30,32,33]. To investigate the impact of 4-substituted Pro residues on collagen thermal stability, triple helices were initially synthesized using monomeric units PPG, PO^4^G, and PFlG [27]. A standard single strand used for triple helix formation typically consists of ten such monomeric units. Comparative analysis of triple helices containing unmodified, 4-hydroxylated, and 4-fluorinated Pro residues revealed a decreasing trend in melting temperature in the order: PFlG (91°C) > PO^4^G (61°C) > PPG (41°C) (Figure 3) [27]. This primarily demonstrated the significance of entropic contribution by EWGs of 4-substituted Pro at Y on the triple helical stability over the water-mediated hydrogen bonding. Further, the structural analysis revealed the favorability of *exo* ring puckering by (4*R*)-Hyp and (4*R*)-Flp at the Y position in the CMPs. The influence of an EWG at (*R*)-diastereoisomeric position enhances the favorability of the *exo* ring pucker that attains the required torsional angles for Y position as a consequence of the gauche effect (Figure 1B) and thereby confers the pre-organization of individual strands for PPII-like helicity to fold into a stable triple helix [27,29,30,50]. Additionally, the analysis also conveys that the greater the electron-withdrawing ability of a substituent at (4*R*), the stronger the gauche effect, thereby increasing the favorability of the *exo* ring pucker. Raines and co-workers extended their investigation with the –OCH_3_ (methoxy) and –Cl (chloro) substituents at 4-C of Pro at Y position [51,52]. They observed a rise in the melting temperatures of triple helices harboring (4*R*)-methoxyproline ((4*R*)-Mop) (70°C) and (4*R*)-chloroproline ((4*R*)-Clp) (52°C) than the melting temperatures of (PPG)_10_ (41°C) and (PO^4^G)_10_ (61°C) and (PPG)_10_, respectively (Figure 3) [51,52]. The (*R*)-methoxy and (*R*)-chloro groups influence the pyrrolidine ring to adopt the *exo* ring pucker, which significantly stabilizes the triple helix. Interestingly, the (4*R*)-Mop, a close analog of (4*R*)-Hyp, reduces the hydration network and stabilizes the triple helix by 9°C compared with (PO^4^G)_10_ [52]. All the 4-substituted Pro ((4*R*)-Flp, (4*R*)-Mop, and (4*R*)-Clp) at Y highlight the importance of the gauche effect, a stereo-electronic effect, presented by the (4*R*)-hydroxyl group in the posttranslationally modified (4*R*)-Hyp on the stability of collagen. (4*R*)-Hyp mediating collagen stability has an enthalpic (formation of hydrogen bonding) and entropic contribution (pre-organization of main chain). Kobayashi and co-workers presented that although the (4*R*)-hydroxyl group reduces the entropic cost of folding, it elevates the cost because of the ability to donate hydrogen bonds [53]. The positional preference of (4*R*)-Hyp-*exo* at Y can be further validated with the incapability of (O^4^PG)_10_ strands assembling into a stable triple helix [54].

Along with the discussed stereo-electronic effects, steric effects also play a role in the conformational folding of single strands into the collagen triple helix by pre-organizing main-chain torsional angles [55]. This was initially explored with (4*S*)-methylproline ((4*S*)-Mep) and (4*S*)-mercaptoproline ((4*S*)-Mcp) [56,57]. Contrary to EWGs, the (*S*)-diastereoisomer of 4-methyl and 4-mercapto groups influences the pyrrolidine ring to adopt an *exo* pucker. The substitution of (4*R*)-Hyp-*exo* in (PO^4^G)_7_ by (4*S*)-Mep and (4*S*)-Mcp reduced the thermal stability by 7°C and 8.5°C, respectively [56,57]. Although their pyrrolidine ring pucker is conformationally preferred for the Y position, the steric effect posed by substituents substantially affects the stability of collagen more than the stereo-electronic effects [56,57]. The profound steric effect of substituents on collagen triple helix stability over the conformational preference of ring pucker for Y position was extensively investigated by Wennemers and co-workers via designing CMPs harboring (*R*)- and (*S*)-diastereoisomers of sterically challenging 4-substituted Pro [58–61]. They incorporated (4*R*)-acetylamidoproline ((4*R*)-Acp), (4*S*)-acetylamidoproline ((4*S*)-Acp), (4*R*)-pivaloylamidoproline ((4*R*)-Pvp), and (4*S*)- pivaloylamidoproline ((4*R*)-Pvp) at Y in the host–guest peptide (PO^4^G)_3_–(PYG)–(PO^4^G)_3_ [59]. The (*R*)- and (*S*)-diastereoisomers of these substituents induce the conformational preference of *exo* and *endo* ring puckers, respectively, as observed in the 4-Hyp residue. The substitution of (4*R*)-Hyp-*exo* with the ring pucker analogs (4*R*)-Acp-*exo* and (4*R*)-Pvp-*exo* in a triple helix can cause destabilization due to steric repulsion. However, the mismatched ring pucker substitution with (4*S*)-Acp-*endo* and (4*S*)-Pvp-*endo* can conformationally stabilize the collagen triple helix (Figure 3) [59]. Therefore, the collagen triple helix can only bear the sterically demanding 4-substituted Pro derivatives at Y, which is equivalent to (4*R*)-Hyp. However, in one of the seminal works by Wennemers and co-workers, they demonstrated that the sterically demanding moieties can be incorporated at the Y position without compromising the conformational stability of the collagen triple helix through click chemistry, such as (4*S*)-triazolyl-Pro derivatives [62]. In summary, previous findings on the correlation between substituents, pyrrolidine ring pucker, and thermal stability by investigating CMPs beautifully highlight the selection of (4*R*)-Hyp at Y in collagen by nature. This section underlines the stereo-electronic and steric effects of an EWG, –OH, that induces the adoption of *exo* ring pucker by (4*R*)-Hyp and nicely accommodates at Y without steric clashes with the neighboring strands.

Interestingly, collagen chains can fold into a stable triple helix when the (4*R*)-Hyp with *exo* ring pucker occupies both the X position and the Y position. From the crystal structures, it was observed that the triple helix adjusts main-chain torsional angles for (4*R*)-Hyp-*exo* to be accommodated at X without disturbing the helicity [63,64]. Moreover, the triple helix with O^4^O^4^G repeats is more stable than that composed of classic PO^4^G repeats [65,66]. A few underlying reasons have been proposed for the stability provided by (4*R*)-Hyp-*exo* at X, such as the interstrand hydrogen bonding [66], interstrand dipole–dipole interaction through C^γ^–OH of (4*R*)-Hyp residues [67], and the reduction in entropic cost for the formation of a water bridge due to the higher level of hydration while the peptide is present in a single-coil state [63]. If the other stereoisomer of hydroxyproline, i.e. (4*S*)-Hyp, accommodates into the X position of the triple helix made of XPG repeats, the triple helix would be less stable, despite (4*S*)-Hyp attaining the favorable endo ring pucker [68]. The reason behind such reduced stability is the transannular hydrogen bond formed between the –OH group of (4*S*)-Hyp and the –CO group of the peptide backbone. This causes a poor pre-organization of the main-chain torsional angles for endo ring pucker and reduces the strength of interstrand hydrogen bonding [68]. The effect of transannular hydrogen bonding was further corroborated through the work of Wennemers and co-workers, who utilized (4*S*)-Amp at X and Y positions [61]. Similar to the (4*S*)-triazolylproline, (4*S*)-ketoproline ((4*S*)-Kep) at the X position in the triple helix was utilized for the bioconjugation [69].

### Evaluating the relationship of the peptide bond proximal to the pyrrolidine ring at Yaa with thermal stability

**Figure 4:**
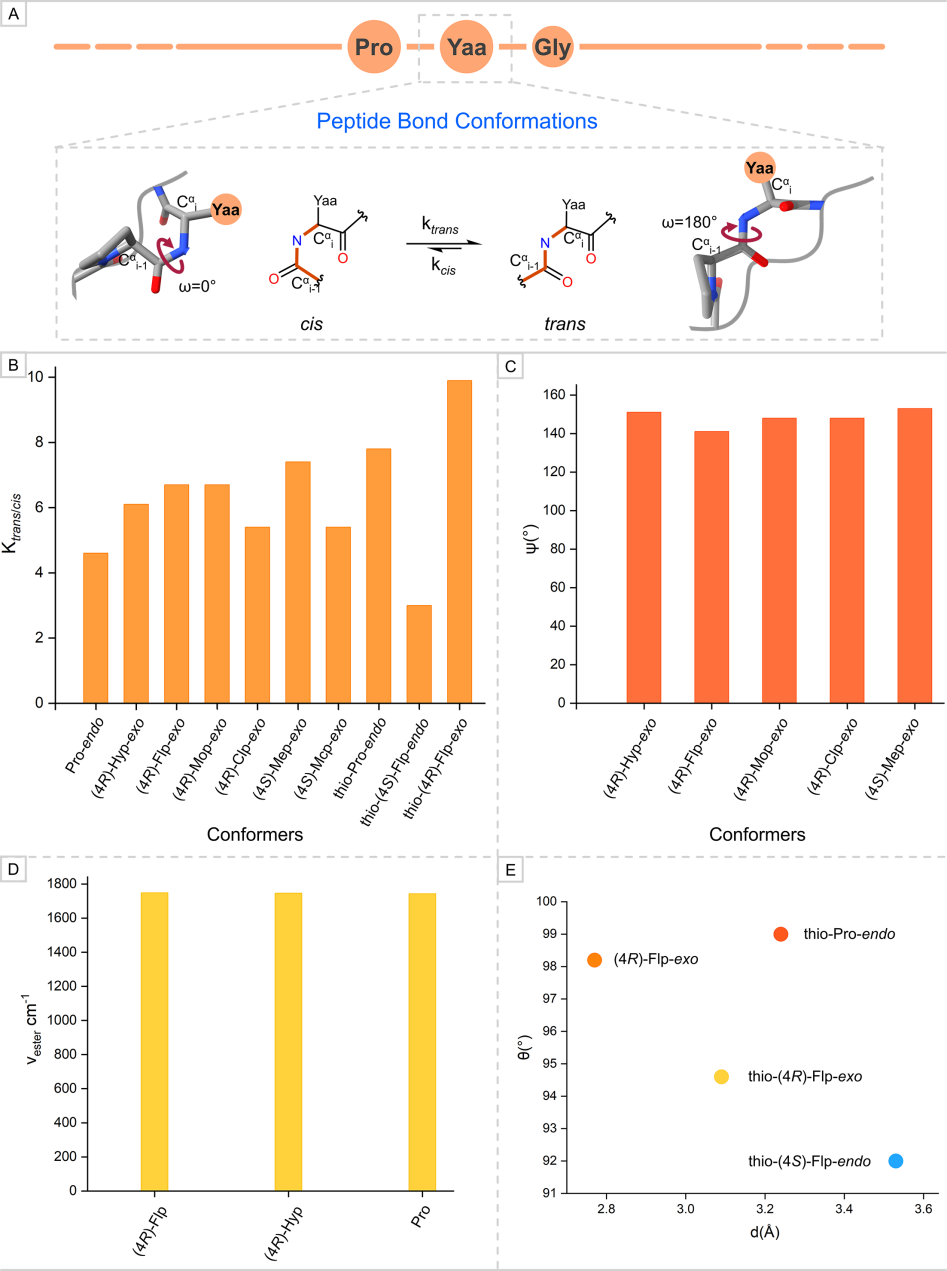
(A) The isomerization of peptide bond with a higher rate for *trans* conformation that is preferred by single strands of collagen. (B) The experimentally calculated isomerization equilibrium constant (K*_trans_*_/_*_cis_*) for proline and its derivatives. (**C**) Values of ψ main-chain torsional angle for proline derivatives. (**D**) The amide bond stretching frequencies of the –CO group observed in IR spectroscopy for proline derivatives. (**E**) The Bürgi–Dunitz trajectory observed in crystallized amide and thioamide conformers of proline derivatives.

Attaining *trans* conformation by peptide bonds is crucial for individual strands of collagen to adopt PPII-like helicity [43,44]. Investigating the factors that influence conformational preference for the *trans* conformer by the peptide bond proximal to Pro and derivatives at Y through CMPs can be challenging. To uncover such factors, Pro derivatives have remained a preferred choice as a model. The primary choice to be analyzed while investigating the attainment of a conformation preferred by a peptide bond is the equilibrium constant (K*_trans_*_/_ *_cis_*). The peptide bond preceding Pro and 4-Hyp residues is isomerized from *cis* to *trans* in a single strand (Figure 4A). Therefore, Pro and 4-Hyp residues must have a higher equilibrium constant for the *trans* conformation. Previous findings suggest a correlation between the *exo* ring pucker and *trans* peptide bond conformation, respectively, especially for (4*R*)-Hyp [70]. This can be validated by comparative analysis of the values of K*_trans_*_/_ *_cis_* for Pro with *endo* ring pucker (Pro-*endo*) and Pro derivatives bearing EWGs at (*R*)-diastereoisomer position with *exo* ring pucker ((4*R*)-Hyp-*exo*, (4*R*)-Flp-*exo*, (4*R*)-Mop-*exo*, and (4*R*)-Clp-*exo*) [30,51,52,70]. Pro-*endo* has a smaller K*_trans_*_/_ *_cis_* (4.6) compared with the (4*R*)-Hyp-*exo* (6.1), (4*R*)-Flp-*exo* (6.7), (4*R*)-Mop-*exo* (6.7), and (4*R*)-Clp-*exo* (5.4) conformers (Figure 4B, Supplementary Table S1) [30,51,52,70]. The analysis demonstrates that the (*R*)-diastereoisomer of EWGs induces the pyrrolidine ring to adopt *exo* ring pucker, which, in turn, prefers the *trans* conformation of the peptide bond. Substituents presenting steric clashes at (4*S*)-diastereoisomer position in Pro ensure the *exo* pyrrolidine ring pucker and the *trans* conformation for peptide bond, as can be seen for (4*S*)-Mep-*exo* and (4*S*)-Mcp-*exo* with K*_trans_*_/_ *_cis_* of 7.4 and 5.4, respectively (Figure 4B, Supplementary Table S1) [56,57]. These values are close to the K_*trans/cis*_ of naturally occurring (4*R*)-Hyp-*exo* conformer (6.1), implicating the correlation between the conformational preferences shown by the pyrrolidine ring and the preceding peptide bond. Another factor associated with the peptide bond conformation and K*_trans_*_/_ *_cis_* is main-chain torsional angle ψ. The value of ψ for (4*R*)-Hyp-*exo*, (4*R*)-Flp-*exo*, (4*R*)-Mop-*exo*, (4*R*)-Clp-*exo*, and (4*S*)-Mep-*exo* is 151°, 141°, 148°, 148°, and 153°, respectively (Figure 4C, Supplementary Table S2) [25,27,30,50–52,56,57]. All conformers bearing substituents that exert stereo-electronic and steric effects tend to adopt ψ torsional angles approaching the optimal value observed in collagen triple helices (~150±9°) [42]. This highlights the role of the pyrrolidine ring pucker of 4-Hyp at the Y position in modulating the ψ angle, thereby pre-organizing individual peptide strands into PPII helical conformation, which is essential for the conformational stability of the collagen triple helix.

The preference for *trans* conformation by peptide bonds in collagen is essential for a charge–transfer interaction, n→π*, to occur between adjacent carbonyl groups (–CO) [30]. The experimental observations for this underlying interaction are the stretching frequency (ν cm^–1^) of the –CO from IR spectroscopy (amide I) and the Bürgi–Dunitz trajectory [70]. The analysis of the carbonyl stretching frequency (ν) revealed only slight differences among (4*R*)-Flp (1748 cm^–1^), (4*R*)-Hyp (1746 cm^–1^), and Pro (1743 cm^–1^) (Figure 4D, Supplementary Table S3) [30,70]. Among these, (4*R*)-Flp exhibited the highest stretching frequency, supporting the notion that the stronger the EWG at the (*R*)-diastereoisomer position, the higher the carbonyl stretching frequency. This trend suggests enhanced redistribution of electron density between the donor–acceptor –CO groups, consistent with the n→π* charge–transfer interaction. The feasibility of the n→π* charge–transfer interaction critically depends on achieving an optimal Bürgi–Dunitz trajectory, characterized by a distance (d) of approximately 3.2 Å and an associated angle (θ) near 109° [45,48]. (4*R*)-Flp-*exo* preferentially adopts the *trans* conformation due to a higher K*_trans_*_/_ *_cis_* ratio, which positions the values of d and θ closer to the optimal Bürgi–Dunitz trajectory. Raines and co-workers explored the relationship between ring pucker, K*_trans_*_/_ *_cis_*, and the Bürgi–Dunitz trajectory by substituting the –CO group in Pro-*endo*, (4*S*)-Flp-*endo*, and (4*R*)-Flp-*exo* conformers with a thioamide (–CS) analog [71]. The resulting thioamide conformers, thio-Pro-*endo* and thio-(4*R*)-Flp-*exo*, exhibited stronger preferences for *trans* conformation, with K*_trans_*_/_ *_cis_* values of 7.8 and 9.9, respectively (Figure 4B). These preferences are reflected in the Bürgi–Dunitz trajectory (d ≈ 3.2 Å, θ ≈ 99°) for thio-Pro-*endo* and (d ≈ 3.1 Å, θ ≈ 94.6°) for thio-(4*R*)-Flp-*exo*, respectively (Figure 4E, Supplementary Table S4). In contrast, the third thioamide conformer, thio-(4*S*)-Flp-*endo*, displayed a weaker preference for *trans* conformation (K*_trans_*_/*cis*_ = 3.0) (Figure 4B, Supplementary Table S1), with a more deviated Bürgi–Dunitz trajectory (d ≈ 3.5 Å, θ ≈ 92°) (Figure 4E, Supplementary Table S4) [71].

Structural studies of collagen using Pro derivatives have demonstrated that the –OH group at (4*R*)-position of Hyp promotes the adaptation of the *exo* ring pucker and *trans* peptide bond conformation. This preference is supported by the isomerization equilibrium constant (K*_trans_*_/_ *_cis_*) and a favorable main-chain torsional angle (ψ). A higher K*_trans_*_/_ *_cis_* ratio, indicative of *trans* peptide bond preference, is essential for achieving an optimal Bürgi–Dunitz trajectory, which, in turn, facilitates the crucial n→π* charge transfer for PPII helicity of individual strands and ultimately the conformational stability of collagen.

## Computational exploration of the influence of conformations preferred by pyrrolidine ring and peptide bond on structural stability of collagen

Since the early efforts to understand collagen stability, computational investigations of Pro and its derivatives—particularly through quantum chemical methods such as DFT—have been instrumental in elucidating the influence of stereo-electronic effects on the pyrrolidine ring and the peptide bond conformations [29,47,48,57,72,73]. Recent studies have demonstrated that the presence of the interplay of these effects in the abundant tripeptide motif, PO^4^G, where (4*R*)-Hyp-*exo* is present at Y [46]. To fully capture the impact of stereo-electronic effects within the context of a collagen triple helix, models involving three collagen strands are preferable and can be effectively studied using the periodic DFT approach [49]. This section reviews key computational findings, ranging from single-residue models to fully periodic triple helix systems, to clarify the role of 4-Hyp in stability.

### Investigating the influence of 4-C substituents on conformational preference for pyrrolidine ring pucker

**Figure 5:**
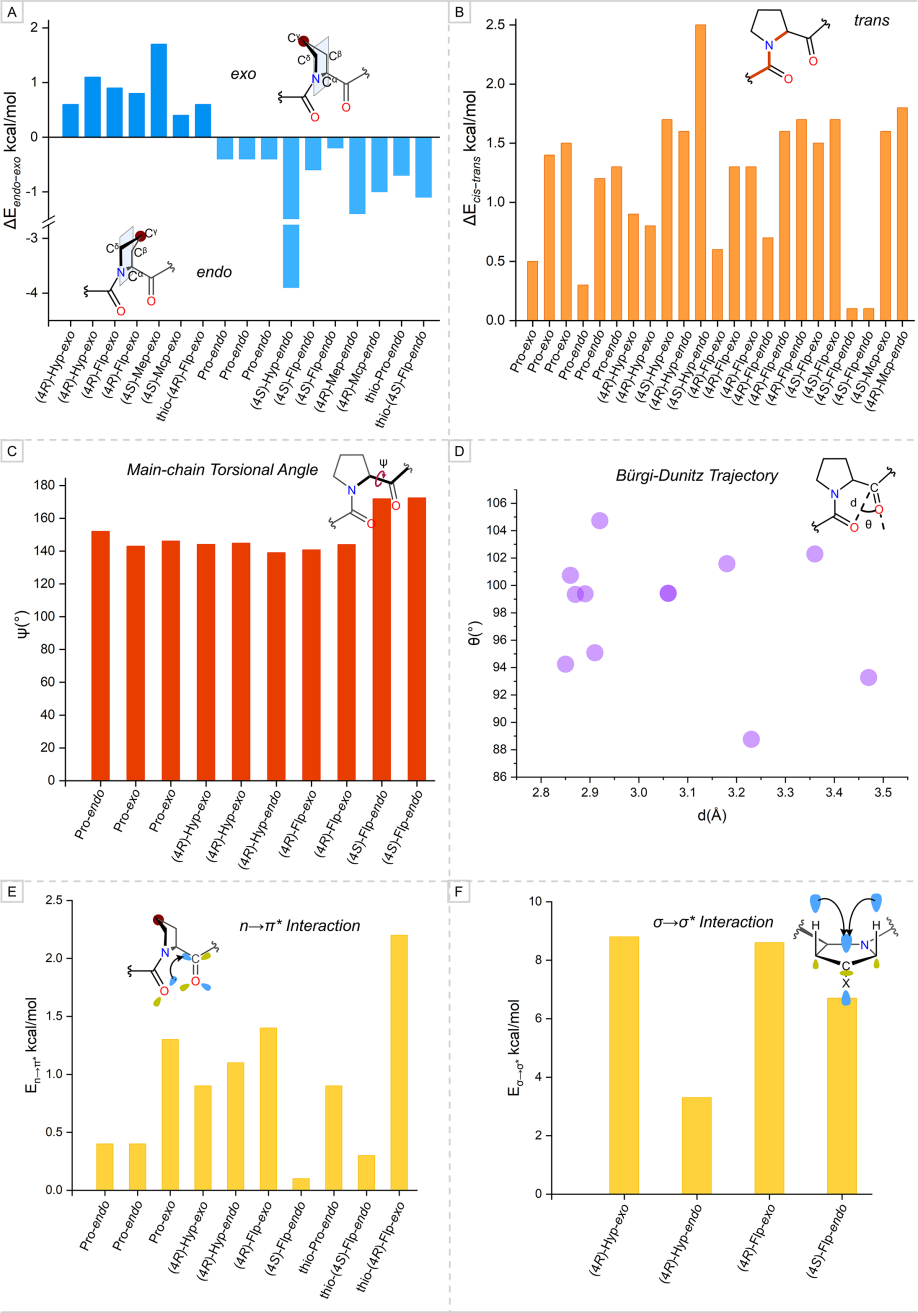
Data were summarized from the density functional theory (DFT)-optimized geometries of proline and its derivatives. (A) The calculated relative energies for ring puckering (ΔE*_endo_*_−_*_exo_*= E[*endo*] – E[*exo*]). The positive and negative energies correspond to the preference of *exo* and *endo* pucker by pyrrolidine ring, respectively. (B) The calculated relative energies for peptide bond conformation (ΔE*_cis_*_−_*_trans_* = E[*cis*] – E[*trans*]). (C) Analysis of ψ main-chain torsional angle. (D) Bürgi–Dunitz trajectory in the peptide backbone representing the major population has values closer to the optimal values for the favorable donor–acceptor interaction. (E) Calculated energies corresponding to the n→π* charge–transfer interaction (E_n→π*_). (F) Calculated energies corresponding to the σ→σ* charge–transfer interaction (E_σ→σ*_).

Previous computational studies have consistently shown that unmodified Pro intrinsically favors the *endo* over the *exo* ring pucker, with calculated relative energy (ΔE*_endo_*_−*exo*_ = E[*endo*] – E[*exo*]) of approximately 0.4 kcal/mol (Figure 5A, Supplementary Table S5). This preference has been reported across various levels of theory, including PBE0/6-31G(d), B3LYP/6-311+G(2d,p), and B3LYP/TZVP (Figure 5A, Supplementary Table S5) [29,47,57]. The posttranslational hydroxylation at the (4*R*)-position alters the χ_1_ ring torsional angle, displacing 4-C above the pyrrolidine ring plane and thereby promoting the *exo* ring pucker. This shift in conformational preference for (4*R*)-Hyp has been corroborated by several studies, with ΔE*_endo_*_−exo_ = 0.6 kcal/mol at the PBE0/6-31G(d) level and 1.1 kcal/mol at the M062X/6–311 + G(d,p) level of theory (Figure 5A, Supplementary Table S5) [46,47]. The conformational preference for the *exo* ring pucker is profound in the case of a stronger electron-withdrawing substituent such as –F. The calculated relative energy (ΔE*_endo_*_−_ *_exo_*) for (4*R*)-Flp at B3LYP/6–311 + G(2d,p) and B3LYP/TZVP levels of theory is 0.9 kcal/mol and 0.8 kcal/mol, respectively [29,57]. Moreover, the thioamide conformer of (4*R*)-Flp prefers the *exo* ring pucker with the relative energy of 0.6 kcal/mol at B3LYP/6–311 + G(2d,p) level of theory (Figure 5A, Supplementary Table S5) [71]. The incorporation of electronegative substituents at (4*R*)- and (4*S*)-position is positively correlated with preference of *exo* and *endo* pyrrolidine ring pucker, respectively. The calculated relative energies in Figure 5A demonstrate this correlation. In contrast, 4-Mep and 4-Mcp bear substituents at the (4*S*)-position that exhibit steric repulsion in collagen triple helix and also preferentially adopt the *exo* ring pucker. The relative energy for (4*S*)-Mep and (4*S*)-Mcp is 1.7 kcal/mol at B3LYP/6–311 + G(2d,p) level and 0.4 kcal/mol at B3LYP/TZVP level of theory, respectively (Figure 5A, Supplementary Table S5) [56,57]. The counterparts of these derivatives that are (4*R*)-Mep and (4*R*)-Mcp demonstrate preference for the *endo* ring pucker (Figure 5A, Supplementary Table S5) [56,57].

### Understanding the conformational preference of peptide bond adjacent to pyrrolidine ring

The main-chain torsional angles (φ, ψ, ω) pre-organize individual strands of collagen triple helix to attain the PPII helicity. In each strand, the peptide bond adopts the *trans* conformation. The two conformations of the peptide bond, *cis* and *trans*, are obtained from its isomerization around the main-chain torsional angle, ω. The analysis of K*_trans_*_/_ *_cis_* has indicated that the *exo* and *endo* ring pucker correlates with the *trans* and *cis* conformations, respectively. However, the computed relative energies (ΔE*_cis_*_−_ *_trans_* = E[*cis*] – E[*trans*]) have unanimously predicted the preference to *trans* conformation by the peptide bond adjacent to Pro and its derivatives, with the range of ΔE*_cis_*_−_ *_trans_* from 0.1 kcal/mol to 2.5 kcal/mol at various levels of theory (Figure 5B, Supplementary Table S6) [29,47,57]. Therefore, the naturally occurring (4*R*)-Hyp-*exo* in collagen prefers to adopt the *trans* conformation of the peptide bond.

### Evaluating the stabilizing charge–transfer interactions

An individual strand of collagen exhibits primarily two types of charge–transfer interactions, n→π* in the peptide backbone and σ→σ* in the pyrrolidine ring. The main-chain torsional angle ψ must approach the optimal value of ~150° in the *trans* conformation of Pro (*vide supra*) and its derivatives for facilitating the favorable n→π* interaction. Previous studies [29,46,47,57] that analyzed the DFT-optimized geometries at various levels of theory have demonstrated that the ψ approaches the optimal value in all Pro and its derivatives except the (4*S*)-Flp-*endo*. The ψ governs the ideal alignment of the Bürgi–Dunitz trajectory in the peptide backbone to maximize the overlap of donor and acceptor orbitals in the stabilizing n→π* interaction [30]. Figure 5D (also see Supplementary Table S7) shows that many reported DFT-optimized structures, including thioamide conformers, exhibit the Bürgi–Dunitz trajectory approaching its ideal values (d ~ 3.2 Å, θ ~ 109°) [29,46,57,71]. This is reflected in the strength of the n→π* interaction (E_n→π*_) (Figure 5E, Supplementary Table S8). Among previously reported conformers, (4*S*)-Flp-*endo* and thio-(4*S*)-Flp-*endo* have Bürgi–Dunitz trajectories that are far away from their ideal value, which, in turn, have reduced the strength of n→π* interaction, with E_n→π*_ of 0.1 kcal/mol and 0.3 kcal/mol, respectively (Figure 5E, Supplementary Table S8) [29,57,71]. Conformers including (4*R*)-Flp-*exo*, (4*R*)-Hyp-*exo*, (4*R*)-Hyp-*endo*, Pro-*exo*, thio-Pro-*endo*, and thio-(4*R*)-Flp-*exo* that exhibit the approximately ideal Bürgi–Dunitz trajectory have stronger n→π* interaction, with E_n→π*_ of 1.4 kcal/mol, 0.9 kcal/mol, 1.1 kcal/mol, 1.3 kcal/mol, 0.9 kcal/mol, and 2.2 kcal/mol, respectively (Figure 5E, Supplementary Table S8) [29,46,57,71]. The thioamide conformer of (4*R*)-Flp-*exo* can significantly donate the electron to the π antibonding orbital (π*) of –CO than the parent conformer. Interestingly, although the Pro-*endo* conformer has the Bürgi–Dunitz trajectory close to the ideal value, the strength of n→π* interaction is weaker, with E_n→π*_ of 0.4 kcal/mol [29,71]. This may imply that the electronegative substituent at 4-C, favorability of ring pucker and peptide bond conformations, and the n→π* interaction are interconnected. Taken together, the *trans* conformation adopted by peptide bond adjacent to pyrrolidine ring fine-tunes the ψ main-chain torsional angle, which, in turn, assists the –CO groups to be aligned with an approximately ideal Bürgi–Dunitz trajectory. This maximizes the overlapping of donor–acceptor orbitals and strengthens the n→π* interaction.

Another charge–transfer interaction, σ→σ*, is the underlying stabilizing interaction for the attained pyrrolidine ring pucker. The initial foundation for this interaction, stabilizing *exo* ring pucker of (4*R*)-Flp-*exo,* was laid by Barone and co-workers [47]. They have demonstrated that the σ bonding orbital of C^β^–H_ax_ and C^δ^–H_ax_ donates an electron to the σ antibonding (σ*) of C^γ^–F. Subsequently, Raines and co-workers have predicted the energy contribution from both the σ→σ* interactions that summed up into a value of E_σ→σ*_ 8.6 kcal/mol and 6.7 kcal/mol for (4*R*)-Flp-*exo* and (4*S*)-Flp-*endo*, respectively (Figure 5F, Supplementary Table S9) [29]. Recently, Mondal and co-workers have predicted the strength of this interaction in two conformers of naturally occurring 4-Hyp residue that are (4*R*)-Hyp-*exo* and (4*R*)-Hyp-*endo*, with E_σ→σ*_ equals to 8.8 kcal/mol and 3.3 kcal/mol, respectively (Figure 5F, Supplementary Table S9) [46]. Therefore, the EWG at (4*R*)-position of the pyrrolidine ring preferentially adopts *exo* ring pucker as this preference is stabilized by stronger σ→σ* interactions.

### Insights from the relevant tripeptide motif

**Figure 6:**
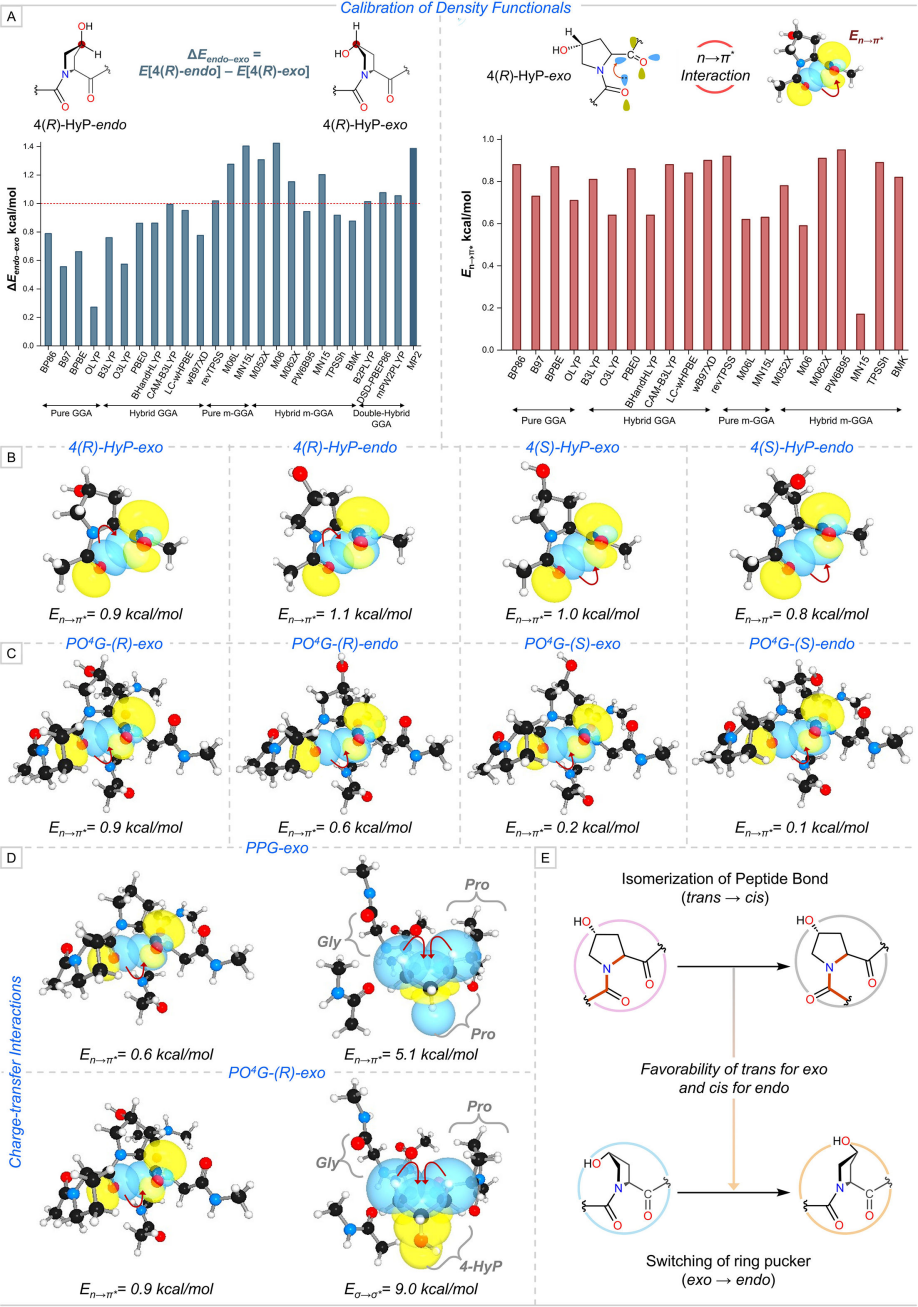
(A) Benchmarking of density functionals against *ab initio* methods for pyrrolidine ring pucker (ΔE_*endo−exo*_) and the n→π* charge–transfer interaction (E_n→π*_) in (*4R*)-Hyp-exo. (B) E_n→π*_ for four conformers of 4-hydroxyproline (4-Hyp) that are (4*R*)-Hyp-*exo*, (4*R*)-Hyp-*endo*, (4*S*)-Hyp-*exo*, and 4*(S*)-Hyp-*endo*. (C) E_n→π*_ for four PO^4^G tripeptide motifs consisting of 4-Hyp conformers, which are PO^4^G-(*R*)-*exo*, PO^4^G-(*R*)-*endo*, PO^4^G-( *S* )-*exo*, and PO^4^G-( *S* )-*endo*. (D) Comparison of strength of charge–transfer interactions between PPG and PO^4^G-( *R* )-*exo* tripeptide motifs. (E) Schematic representation for the interconnection between the attainment of conformations by the pyrrolidine ring and the peptide bond. Part of the figure has been used with permission from the Elsevier [46].

Computational investigations of Pro and its derivatives have been reported by different research groups utilizing various DFT methods, which can be seen in previous subsections. This can make a person uncertain about the DFT method to be used to study collagen stability. In a recent study, Mondal and co-workers calibrated 24 DFT functionals against *ab initio* methods (MP2 and DLPNO-CCSD(T)) on 4-Hyp for the primary factors that stabilize collagen, which are conformational preference of pyrrolidine ring and the n→π* charge–transfer interaction (Figure 6A) [46]. Consequently, they proposed the Minnesota functional M062X as a choice of method. From a thorough analysis of the n→π* interaction on four conformers of 4-Hyp, the anomaly in a single residue model was discovered that the non-natural conformers (4*R*)-Hyp-*endo* and (4*S*)-Hyp-*exo* have E_n→π*_ relatively larger by 0.2 kcal/mol and 0.1 kcal/mol, respectively, than the natural conformer (4*R*)-Hyp-*exo* (Figure 6B) [46]. To overcome this, a relevant and abundant tripeptide motif of PO^4^G to computationally study the stability of the collagen triple helix was established. The model was validated by incorporating four conformers of 4-Hyp into the Y position in the tripeptide motif and analyzing the n→π* interaction around those conformers [46]. This analysis demonstrated that the strength of the n→π* interaction around 4-Hyp is more in the tripeptide motif harboring the natural conformer (4*R*)-Hyp-*exo* (PO^4^G-(*R*)-*exo*), with E_n→π*_ of 0.9 kcal/mol (Figure 6C). The electronic structure investigation was further extended to reveal the impact of prolyl-4-hydroxylation on the two primary charge–transfer interactions (*vide supra*). The hydroxylation of Pro at Y strengthens the n→π* interaction by 0.3 kcal/mol and thereby the conformational preference for the *trans* conformer of the peptide bond. Additionally, the stabilization of the attained *exo* pucker by the pyrrolidine ring is increased by ~4.0 kcal/mol as a result of a stronger σ→σ* interaction occurring in PO^4^G-(*R*)-*exo* (Figure 6D).

In this study, our group established a clear correlation between the pyrrolidine ring pucker and the peptide bond conformation [46]. A two-dimensional (2D) scan was performed on the torsional angles of the peptide bonds preceding and succeeding the natural (4*R*)-Hyp-*exo* conformer. The resulting conformational energy landscape encompasses all the conformers, including the *trans*/*trans* and *cis*/*cis* configurations, where both preceding and succeeding peptide bonds adopt the *trans* and *cis* conformation, respectively. The isomerization trajectory of the adjacent peptide bonds was monitored across the conformational energy landscape. As the system transitions from *trans*/*trans* configuration toward the highest energy point (i.e. the transition state), the pyrrolidine ring of (4*R*)-Hyp flattens, shifting from *exo* pucker toward a planar conformation. Subsequently, the pyrrolidine ring adopts the *endo* ring pucker as the peptide bonds approach the *cis*/*cis* configuration [46]. This analysis reveals a coupled conformational mechanism, in which the pyrrolidine ring pucker interconverts in synchronization with the peptide bond isomerization (Figure 6E). The evolution of electronic structure was examined along this transition path—from the *exo* ring pucker with *trans*/*trans* peptide bond configuration to the *endo* ring pucker with *cis*/*cis* peptide bond configuration [46]. Within the peptide backbone, the n→π* interaction—the interaction between the adjacent –CO groups in the *trans*/*trans* configuration—rapidly diminishes once the system deviates from this conformation. In contrast, the σ→σ* interactions involving C^β^–H_ax_/C^δ^–H_ax_ and C^γ^–OH reduce more gradually during the transition and are completely lost in the *cis*/*cis* configuration, which adopts the endo ring pucker. In this final state, the conformer is instead stabilized by alternative σ→σ* interactions involving C^β^–H_eq_/C^δ^–H_eq_. This quantitatively establishes the interplay between the different stereo-electronic effects.

## Understanding stability through the modeling of periodic structure of collagen

**Figure 7:**
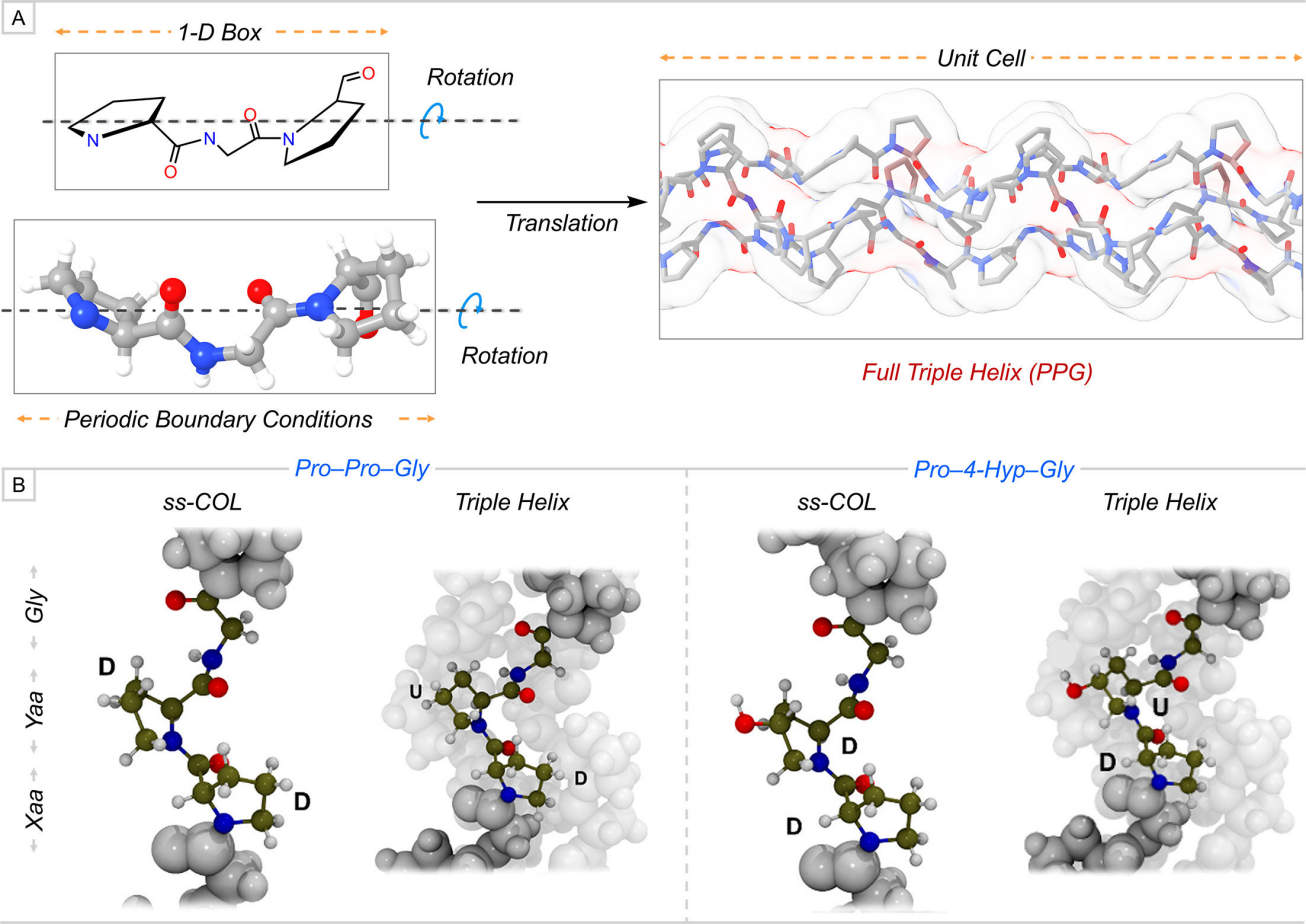
(A) Schematic representation to model a full triple helix from a tripeptide motif by invoking roto-translational symmetry and periodic boundary conditions using periodic density functional theory (DFT) approach. (B) ss-COL and triple helix models consist of PPG (Left) and PO^4^G (right) tripeptide motifs. D and U represent the down (*endo*) and up (*exo*) pyrrolidine ring pucker, respectively. Part of the figure has been used with permission from the American Chemical Society [49].

Individual strands of collagen comprise a repeating tripeptide motif in the form of XYG, which fold into a triple helical structure. Computational studies focused on either single residues or tripeptide motifs can effectively capture local stabilizing stereo-electronic effects within a single strand of collagen. However, such models may overlook additional stabilizing interactions inherent to the full structure, such as interstrand hydrogen bonding. As collagen exhibits periodicity, a quantum chemical method employing periodic DFT is well suited to evaluate stability on a more realistic triple helical structure. Recently, Ugliengo and co-workers employed the periodic DFT approach to investigate the critical role of 4-Hyp in the stability of collagen [49]. Single-strand (ss-COL) and triple helix models consist of tripeptide motifs such as PPG and PO^4^G that were prepared by invoking the roto-translational symmetry and periodic boundary conditions for a 1D periodic structure (Figure 7A) [49,74,75]. ss-COL and triple helix structures were investigated to evaluate the interconnection between the pyrrolidine ring pucker of Pro and 4-Hyp at X and Y, and the stability. In the case of ss-COL, the model in which both the Pro residues present at X and Y adopt the *endo* (D) ring pucker is stable by 1.3 kJ/mol than the model that has Pro residues that preferentially adopt *endo* and *exo* (U) ring pucker at X and Y, respectively (Figure 7B). However, the conformational preference of ring pucker for Pro at Y shifts to *exo*, which stabilizes the triple helix by 1.6 kJ/mol than the triple helix consisting of Pro residues with the *endo* ring pucker at both Xaa and Yaa positions (Figure 7B). Similar observations were reported in the case of ss-COL and the triple helix that consists of PO^4^G. The (4*R*)-Hyp adopts the endo ring pucker in the stable ss-COL, whereas it adopts the exo ring pucker in the stable triple helix (Figure 7B). The binding energy for triple helices comprising PPG and PO^4^G was also calculated, which demonstrated that the PO^4^G-containing triple helix is stable by ~3 kJ/mol than the triple helix comprising PPG. They proposed that the interstrand binding energy has a significant contribution from the London dispersion interaction, which can be increased with the incorporation of 4-Hyp at Y. Additionally, a small amount of H-bond energy was calculated in triple helices, indicating a weaker interstrand hydrogen bonding network [49].

## Conclusion and outlook

This review consolidates the experimental and computational approaches that have been extensively employed to investigate the factors that make 4-Hyp indispensable in the structural stability of collagen. It highlights the keen observations made from the CMPs and single amino acid residues to understand that Pro and (4*R*)-Hyp residues adopting *endo* and *exo* ring pucker at X and Y, respectively, and the peptide bond attaining *trans* conformation are necessary for individual strands to assemble into a collagen triple helix. Moreover, the computational studies utilizing the power of DFT to evaluate the influence of –OH at (4*R*)- or (4*S*)-position on the conformational preference of pyrrolidine ring, the underlying charge–transfer interactions stabilizing *exo* and *endo* pyrrolidine ring puckers and *trans* peptide bond conformation, and the correlation between the ring pucker and peptide bond conformation are summarized. Additionally, we have highlighted the application of the periodic DFT approach to explore the effect of 4-Hyp on the structure and stability of collagen. In addition to summarizing the known previous literature, this review opens several avenues to pursue further research to substantially understand the sequence–structure relationship in collagen. Among the possible tripeptide motifs, only PO^4^G has been explored so far. The impact of different amino acids occupying the X position in the XO^4^G tripeptide motif on the stabilizing stereo-electronic effects exhibited by 4-Hyp at Y and ultimately on the local and global structural stability of collagen still remained unknown. The electronic structure of the rare PTM of Pro at X, i.e. 3-Hyp, is yet to be investigated to evaluate its role in collagen. Moreover, the influence of stereo-electronic effects on collagen stability has been limited to a single strand, which needs to be extended to a folded triple helix to capture the insights at the macromolecular level. In summary, the review presents the detailed approaches employed to investigate the role of 4-Hyp in collagen stability and intrigues one to pursue open questions and expand the horizon of the current knowledge regarding the sequence–structure relationship in collagen.

## Supplementary material

online supplementary material 1.

## Open Access

This article has been published open access under our Subscribe to Open programme, made possible through the support of our subscribing institutions, learn more here: https://portlandpress.com/pages/open_access_options_and_prices#conditional
