# Supplementary material for: Molecular foundations of collagen triple helical assembly: the central role of prolyl-4-hydroxylation
Source: Biochem J. 2026 Jan 8;483(2):BCJ20253467. doi: 10.1042/BCJ20253467 (PMC12862961; doi:10.1042/BCJ20253467)
Supplement: online supplementary material 1. [file bcj-483-2-BCJ20253467-s001.docx]

*Supplementary Information*

# Molecular Foundations of Collagen Triple Helical Assembly: The Central Role of Prolyl-4-Hydroxylation

Ashutosh Joshi^1^, Bhaskar Mondal*^2^, Trayambak Basak*^1^

1School of Biosciences and Bioengineering, Indian Institute of Technology Mandi, Mandi, Himachal Pradesh-175075, India

2 School of Chemical Sciences, Indian Institute of Technology Mandi, Mandi, Himachal Pradesh-175075, India

*Corresponding Authors’ Email: [bhaskarmondal@iitmandi.ac.in](mailto:bhaskarmondal@iitmandi.ac.in) (BM); trayambak@iitmandi.ac.in(TB)

Table S1. The isomerization equilibrium constant (K*trans*/*cis*) of conformers of proline derivatives from various experimental studies.

| **S.No.** | **Conformer** | **K*trans*/*cis*** |
| --- | --- | --- |
| **1** | Pro-*endo* | 4.6 |
| **2** | 4(*R*)-HyP-*exo* | 6.1 |
| **3** | 4(*R*)-FlP-*exo* | 6.7 |
| **4** | 4(*R*)-MoP-*exo* | 6.7 |
| **5** | 4(*R*)-ClP-*exo* | 5.4 |
| **6** | 4(*S*)-MeP-*exo* | 7.4 |
| **7** | 4(*R*)-McP-*exo* | 5.4 |
| **8** | thio-Pro-*endo* | 7.8 |
| **9** | thio-4(*S*)-FlP-*endo* | 3 |
| **10** | thio-4(*R*)-FlP-*exo* | 9.9 |

Table S2. The ψ main-chain torsional angles of conformers of proline derivatives from various experimental studies.

| **S.No.** | **Conformer** | **ψ (°)** |
| --- | --- | --- |
| **1** | 4(*R*)-HyP-*exo* | 151 |
| **2** | 4(*R*)-FlP-*exo* | 141 |
| **3** | 4(*R*)-MoP-*exo* | 148 |
| **4** | 4(*R*)-ClP-*exo* | 148 |

Table S3. The stretching frequency of –CO group of conformers of proline derivatives from various experimental studies.

| **S.No.** | **Conformer** | **νester (cm-1)** |
| --- | --- | --- |
| **1** | 4(*R*)-FlP | 1748 |
| **2** | 4(*R*)-HyP | 1746 |
| **3** | Pro | 1743 |

Table S4. The Bürgi-Dunitz trajectory between two subsequent –CO groups of conformers of proline derivatives from various experimental studies.

| **S.No.** | **Conformer** | **d (Å)** | **θ (°)** |
| --- | --- | --- | --- |
| **1** | 4(*R*)-FlP-*exo* | 2.77 | 98.2 |
| **2** | thio-Pro-*endo* | 3.24 | 99 |
| **3** | thio-4(*S*)-FlP-*endo* | 3.53 | 92 |
| **4** | thio-4(*R*)-FlP-*exo* | 3.09 | 94.6 |

Table S5. The relative energy calculated for two pyrrolidine ring puckers of conformers of proline derivatives through various DFT methods.

| **S.No.** | **Conformer** | **ΔE*endo*−*exo* kcal/mol** |
| --- | --- | --- |
| **1** | 4(*R*)-HyP-*exo* | 0.6 |
| **2** | 4(*R*)-HyP-*exo* | 1.1 |
| **3** | 4(*R*)-FlP-*exo* | 0.9 |
| **4** | 4(*R*)-FlP-*exo* | 0.8 |
| **5** | 4(*S*)-MeP-*exo* | 1.7 |
| **6** | 4(*S*)-McP-*exo* | 0.4 |
| **7** | thio-4(*R*)-FlP-*exo* | 0.6 |

| **8** | Pro-*endo* | -0.4 |
| --- | --- | --- |
| **9** | Pro-*endo* | -0.4 |
| **10** | Pro-*endo* | -0.4 |
| **11** | 4(*S*)-HyP-*endo* | -3.9 |
| **12** | 4(*S*)-FlP-*endo* | -0.6 |
| **13** | 4(*S)*-FlP-*endo* | -0.2 |
| **14** | 4(*R*)-MeP-*endo* | -1.4 |
| **15** | 4(*R*)-McP-*endo* | -1 |
| **16** | thio-Pro-*endo* | -0.7 |
| **17** | thio-4(*S*)-FlP-*endo* | -1.1 |

Table S6. The ψ main-chain torsional angles observed in the optimized geometries of conformers of proline derivatives through various DFT methods.

| **S.No.** | **Conformer** | **ψ (°)** |
| --- | --- | --- |
| **1** | Pro-*exo* | 0.5 |
| **2** | Pro-*exo* | 1.4 |
| **3** | Pro-*exo* | 1.5 |
| **4** | Pro-*endo* | 0.3 |
| **5** | Pro-*endo* | 1.2 |
| **6** | Pro-*endo* | 1.3 |
| **7** | 4(*R*)-HyP-*exo* | 0.9 |
| **8** | 4(*R*)-HyP-*exo* | 0.8 |
| **9** | 4(*S*)-HyP-*exo* | 1.7 |
| **10** | 4(*R*)-HyP-*endo* | 1.6 |

| **11** | 4(*S*)-HyP-*endo* | 2.5 |
| --- | --- | --- |
| **12** | 4(*R*)-FlP-*exo* | 0.6 |
| **13** | 4(*R*)-FlP-*exo* | 1.3 |
| **14** | 4(*R*)-FlP-*exo* | 1.3 |
| **15** | 4(*R*)-FlP-*endo* | 0.7 |
| **16** | 4(*R*)-FlP-*endo* | 1.6 |
| **17** | 4(*R*)-FlP-*endo* | 1.7 |
| **18** | 4(*S*)-FlP-*exo* | 1.5 |
| **19** | 4(*S*)-FlP-*exo* | 1.7 |
| **20** | 4(*S*)-FlP-*endo* | 0.1 |
| **21** | 4(*S*)-FlP-*endo* | 0.1 |
| **22** | 4(*S*)-McP-*exo* | 1.6 |
| **23** | 4(*R*)-McP-*endo* | 1.8 |

Table S7. The Bürgi-Dunitz trajectory between two subsequent –CO groups observed in the optimized geometries of conformers of proline derivatives through various DFT methods.

| **S.No.** | **Conformer** | **d (Å)** | **θ (°)** |
| --- | --- | --- | --- |
| **1** | Pro-*endo* | 3.06 | 99.43 |
| **2** | Pro-*endo* | 3.06 | 99.43 |
| **3** | Pro-*exo* | 2.87 | 99.35 |
| **4** | 4(*R*)-HyP-*exo* | 2.89 | 99.39 |
| **5** | 4(*R*)-HyP-*exo* | 2.85 | 94.25 |
| **6** | 4(*R*)-HyP-*endo* | 2.91 | 95.09 |
| **7** | 4(*R*)-HyP-*endo* | 2.92 | 104.73 |

| **8** | 4(*R*)-FlP-*exo* | 2.86 | 100.74 |
| --- | --- | --- | --- |
| **9** | 4(*S*)-FlP-*endo* | 3.23 | 88.76 |
| **10** | thio-Pro-*endo* | 3.36 | 102.3 |
| **11** | thio-4(*S*)-FlP-*endo* | 3.47 | 93.28 |
| **12** | thio-4(*R*)-FlP-*exo* | 3.18 | 101.59 |

Table S8. The stabilization energy due to the n→π* charge-transfer interaction at peptide backbone observed in the optimized geometries of conformers of proline derivatives through various DFT methods.

| **S.No.** | **Conformer** | **En→π* kcal/mol** |
| --- | --- | --- |
| **1** | Pro-*endo* | 0.4 |
| **2** | Pro-*endo* | 0.4 |
| **3** | Pro-*exo* | 1.3 |
| **4** | 4(*R*)-HyP-*exo* | 0.9 |
| **5** | 4(*R*)-HyP-*endo* | 1.1 |
| **6** | 4(*R*)-FlP-*exo* | 1.4 |
| **7** | 4(*S*)-FlP-*endo* | 0.1 |
| **8** | thio-Pro-*endo* | 0.9 |
| **9** | thio-4(*S*)-FlP-*endo* | 0.3 |
| **10** | thio-4(*R*)-FlP-*exo* | 2.2 |

Table S9. The stabilization energy due to the σ→σ* charge-transfer interaction in pyrrolidine ring observed in the optimized geometries of conformers of proline derivatives through various DFT methods.

| **S.No.** | **Conformer** | **Eσ→σ* kcal/mol** |
| --- | --- | --- |
| **1** | 4(R)-HyP-exo | 8.8 |
| **2** | 4(R)-HyP-endo | 3.3 |
| **3** | 4(R)-FlP-exo | 8.6 |
| **4** | 4(S)-FlP-endo | 6.7 |
